# *Arabis alpina*: A perennial model plant for ecological genomics and life‐history evolution

**DOI:** 10.1111/1755-0998.13490

**Published:** 2021-09-07

**Authors:** Stefan Wötzel, Marco Andrello, Maria C. Albani, Marcus A. Koch, George Coupland, Felix Gugerli

**Affiliations:** ^1^ Institute of Ecology, Evolution and Diversity Goethe University Frankfurt and Senckenberg Biodiversity and Climate Research Centre Frankfurt (Main) Germany; ^2^ Institute for the Study of Anthropic Impacts and Sustainability in the Marine Environment National Research Council, CNR‐IAS Rome Italy; ^3^ Institute for Plant Sciences University of Cologne Cologne Germany; ^4^ Biodiversity and Plant Systematics Centre for Organismal Studies (COS) Heidelberg University Heidelberg Germany; ^5^ Department of Plant Development Biology MPI for Plant Breeding Research Cologne Germany; ^6^ WSL Swiss Federal Research Institute Birmensdorf Switzerland

**Keywords:** arctic–alpine environment, Brassicaceae, local adaptation, perennial, selfing

## Abstract

Many model organisms were chosen and achieved prominence because of an advantageous combination of their life‐history characteristics, genetic properties and also practical considerations. Discoveries made in *Arabidopsis thaliana*, the most renowned noncrop plant model species, have markedly stimulated studies in other species with different biology. Within the family Brassicaceae, the arctic–alpine *Arabis alpina* has become a model complementary to *Arabidopsis thaliana* to study the evolution of life‐history traits, such as perenniality, and ecological genomics in harsh environments. In this review, we provide an overview of the properties that facilitated the rapid emergence of *A*. *alpina* as a plant model. We summarize the evolutionary history of *A*. *alpina*, including genomic aspects, the diversification of its mating system and demographic properties, and we discuss recent progress in the molecular dissection of developmental traits that are related to its perennial life history and environmental adaptation. From this published knowledge, we derive open questions that might inspire future research in *A*. *alpina*, other Brassicaceae species or more distantly related plant families.

## 1 INTRODUCTION

Describing and understanding the overwhelming diversity of life forms depends on the establishment of model species as common study platforms and references (Box 1). By focusing on a single organism with practical advantages, a wealth of knowledge can be generated. This has led to tremendous advances in the understanding of fundamental principles in many disciplines of biology (reviewed, e.g., in Müller & Grossniklaus, 2010). In plant sciences, *Arabidopsis thaliana* has served as a widely used model for several decades (Weigel, 2012). It combines many key characteristics of a successful plant model: short life cycle, wide distribution, variation in life‐history traits, self‐compatibility, small genome size, genetic tractability, facile transformation and easy cultivation. However, no model can cover all areas of biological interest. Consequently, the number of dedicated plant model species has increased continuously (Cesarino et al., 2020; Kane et al., 2013; Koenig & Weigel, 2015), which will benefit future research in many fields (Pyhäjärvi & Mattila, 2021).

BOX 1 **How to become and be a plant model species**

There are numerous aspects required to make a plant species become a model (e.g., for evolutionary biology). Commonly, such a model species is originally adopted to study a very particular biological question in detail, and hence a specific trait may be decisive for choosing the appropriate organism for the given study. Subsequently, such a case model may be further elaborated and eventually serves as a reference for many other taxa and, hence, is commonly recognized as a model species (Cesarino et al., 2020).

Nonetheless, there are some preferential characteristics that enhance the chance for a species to emerge as a model, such as distinct life‐history characteristics, particular traits or trait variants, wide occurrence under various environmental conditions, simple/short life cycle, easy experimental handling, small genome and possibly close relatedness—but with complementary traits—to other model species. The last helps explain why many Brassicaceae nowadays serve as model systems that emerged in the backwash of *Arabidopsis thaliana*. But in the end, it is often the gathering of a wide array of experimental and genomic resources, such as a high‐quality genome and transcriptome, functional annotation, phylogenetic characterization, etc., which pave the way for a species to make it into the illustrious circle of model species.

Recently recognized, “post‐*Arabidopsis thaliana*” model species (excluding crops) and their particular trait peculiarities are: *Arabidopsis halleri* for its heavy‐metal tolerance (Hanikenne et al., 2008) and as an outcrossing complement to *Arabidopsis thaliana* (reviewed in Honjo & Kudoh, 2019), *Arabidopsis lyrata* for shifts in mating system (Mable et al., 2005), adaptation (Kemi et al., 2013) and population genomics (Hämälä & Savolainen, 2019), *Arabidopsis arenosa* for evolutionary ecological genomics (Kolář et al., 2016) and, more specifically, polyploidy associated with hybridization (Monnahan et al., 2019; Yant & Bomblies, 2017), and *Cardamine hirsuta* to study compound leaf development and seed dispersal (Hay et al., 2014). Beyond the Brassicaceae, the liverwort *Marchantia polymorpha* (Bowman, 2016) and the grass *Brachypodium distachyon* (Scholthof et al., 2018), but also the first fully sequenced tree species *Populus trichocarpa* (Yang et al., 2010), are, among others, well‐recognized model species for diverse reasons.

So why does *Arabis alpina* qualify for being a model species? This species combines many key aspects listed above, among others a high‐quality genome (Jiao et al., 2017; Willing et al., 2015), a broad distribution in contrasting habitat conditions including elevation, easy maintenance and propagation under glasshouse conditions, and the possibility for genetic modification. At the same time, there are other reasons, such as contrasting traits to those found in *Arabidopsis thaliana*, but also challenges in various aspects, which make *A*. *alpina* a species which will attract further research: perenniality, variation in mating system among populations, variation in flowering behaviour, potential for adventitious rooting, nonsegregating pericentromeric regions and adaptation to arctic–alpine environment to name a few. Irrespective of these scientific reasons, a certain, but anecdotal degree of chance has contributed to *A*. *alpina* becoming a prominent species in the study of ecological genomics.

A missing aspect in the formerly established model systems (see Box 1) was how alpine and arctic environments may relate to the evolution of a perennial life history. During the past 15 years, *Arabis alpina* L. (Brassicaceae), the Alpine rockcress, has been established as a complementary model species for ecological genomics and life‐history evolution under harsh abiotic conditions. While evolutionary research on *A*. *alpina* had started nearly 60 years ago with taxonomic investigations by Hedberg (1962) comparing populations from Africa and Scandinavia, it was only about 50 years later that phylogeographical studies inferred the species' Pleistocene and postglacial history (Ehrich et al., 2007; Koch et al., 2006). Shortly after, experimental studies highlighted that the differential breakdown of self‐incompatibility has led to populations with varying degrees of self‐compatibility (Ansell et al., 2008). These initial studies paved the way for further in‐depth research on the many aspects of life‐history evolution in *A*. *alpina* reviewed here.

*Arabis alpina* is diploid, with a base chromosome number of *n* = 8, and its karyotype resembles the putative ancestral state of the Brassicaceae, which is in contrast to its *Arabidopsis* relatives with *n* = 5. The species can equally be genetically manipulated by *Agrobacterium*‐mediated transformation and, hence, is amenable to the toolkit of molecular biology so that the species has developed into a model system for addressing the molecular mechanisms of perenniality (Wang, Farrona, et al., 2009). With the release of a first reference genome assembly (Willing et al., 2015), comparative genomic analyses have become possible, and similar to the development in the genus *Arabidopsis*, studies have widened to include other species from the genus *Arabis* (Kiefer et al., 2017).

**FIGURE 1:**
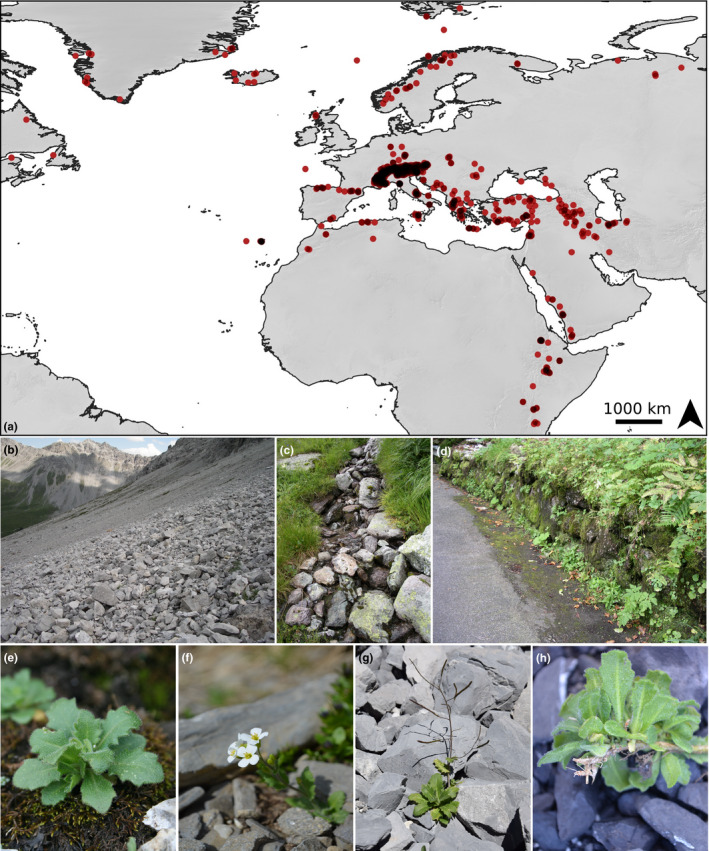
Sampled populations of *Arabis alpina* cover large parts of its global range (cf. Ansell et al., 2011) and indicate a focus on the European Alps (a). Points represent coordinates of published populations (data listed in the Appendix S1), and darker red colour indicates higher density of studies in given areas. Top row photos show typical alpine habitats of *A. alpina*: calcareous scree (b), moist habitats along small creeks (c), and areas of nutrient sinks such as road verges (d). Bottom row photos illustrate morphology of intermediately sized vegetative (e), flowering (f) and fruiting (g) individuals. The close‐up photo (h) shows adventitious roots growing from a mature shoot at the base of vegetative rosettes Photos: F. Gugerli and S. Wötzel; map made with Natural Earth

The arctic–alpine *A*. *alpina* has a wide geographical distribution in the European Alps, Spain, Arabia and East Africa, and extends into Scandinavia, southern Greenland and northeastern most parts of North America (cf. Ansell et al., 2011; Figure 1). Corresponding to its broad range, habitats of *A*. *alpina* span a wide ecological amplitude and elevation. Typically, plants occur on calcareous scree slopes and rocky debris, where they can persist by elongating shoots between the unstable substrate. However, individuals also thrive in more sheltered areas where nutrients often accumulate due to dung deposition of cattle, wild ungulates or birds, and they can tolerate very moist conditions in moss‐dominated communities along creeks or ravines (Figure 1). Many of these habitats are transient and can restrict the lifespan of individual plants, which exemplifies the need for developmental flexibility. Given its wide distribution and broad ecological niche within an arctic–alpine setting, *A*. *alpina* complements the aforementioned model species by expanding the breadth of environmental conditions for studying ecological genomics and life‐history evolution near to the extremes of plant occurrence, both in latitude and in elevation.

Here, we provide an overview of current knowledge on *A*. *alpina* and introduce the various types of resources recently developed. We summarize the species' phylogeographical and evolutionary systematic history and discuss its use as a model for studies of adaptation, mating system evolution and the dissection of complex developmental traits. Moreover, we include the latest progress in understanding its perennial life history and the molecular and physiological underpinnings of it, and finally provide perspectives on potential future research.

## 2 EVOLUTION, SYSTEMATICS AND PHYLOGEOGRAPHY

Tribe Arabideae, which includes *Arabis alpina*, is one of the most prominent tribes within the family Brassicaceae. It is a monophyletic assemblage with roughly 545 species distributed among 18 accepted genera, and convergence of morphological traits and trait complexes is found in all main lineages (Walden, German, et al., 2020). Based on recent cytogenetic evidence, Arabideae has been considered one of the early emerging tribes among the evolutionary lineages described in Brassicaceae, that is probably basal to all lineages except Aethionemeae (Walden, Nguyen, et al., 2020). However, this finding contradicts phylogenomic analyses (Kiefer et al., 2019; Mabry et al., 2020; Nikolov et al., 2019; Walden, German, et al., 2020) that placed the tribe close to Brassicaceae evolutionary lineage II (Box 1, Figure B1a). Future phylogenomic analyses considering genomic context, syntenic regions and cytogenetic blocks may provide the link between these contradicting findings. Despite this uncertainty, stem group and crown group ages of the tribe can be roughly estimated at 20 and 18 million years ago, respectively (Huang et al., 2020; Walden, German, et al., 2020).

Genus *Arabis*, which forms the core of tribe Arabideae, is a well‐studied para‐ and polyphyletic set of ~100 species (Karl & Koch, 2013), for which *A*. *alpina* serves as the type species. However, the genus at present does not form a monophyletic group and will probably undergo further taxonomic revision. Even among the taxa currently considered as closely related to *A. alpina*, including *A*. *caucasica* that is often used as an ornamental plant, there is ample taxonomic uncertainty that must still be resolved. Nevertheless, this taxonomic group encloses species with a great variety of life‐history characteristics that may serve as comparative study systems. A detailed account of the systematic and taxonomic state of knowledge—and uncertainty—is given in Box 2.

BOX 2 **Evolution and systematics of *Arabis***

**Evolution of tribe Arabideae**

Among the 18 genera and 545 species within tribe Arabideae (Walden, German, et al., 2020), 63% are neopolyploids, and its mean net diversification rate is more than three times higher than the family mean (Huang et al., 2020), highlighting the evolutionary dynamics of this lineage. Research in the past two decades has resulted in a well‐resolved phylogenetic tree that demonstrates paraphyly of the genus *Arabis* and established a new systematic concept (Karl & Koch, 2013). Multiple radiations in various clades involved a split between lowland annual and montane/alpine perennial sister species, in which increased speciation rates occurred frequently.

The centre of origin of tribe Arabideae is the Irano‐Turanian region (Figure B1), which ranges from the eastern Mediterranean and Saharo‐Arabian regions to the Tian Shan and Pamir Mountains (for a detailed review on this floristic region, see Manafzadeh et al., 2017). From there, the different clades independently colonized the temperate and alpine mountain regions of the world (Karl & Koch, 2013). Generally, numerous morphological trait characteristics have evolved convergently and are often linked, for example, in annuals vs. perennials. In contrast to perennial species, annuals exhibit a complete selfing syndrome, have wider and lowland distribution ranges, did not undergo subsequent radiations, and exhibit lower genetic variation that might, among others, result from the selfing syndrome (Karl & Koch, 2013, 2014).

**Systematic and taxonomic considerations of genus *Arabis***

The type species of the genus *Arabis* is represented by the Linnean holotype of *A*. *alpina* (Species Plantarum 2: 664; Linnaeus, 1753). However, if future taxonomic work aims for a monophyletic genus *Arabis*, then most of the species contained in the genus at present will have to be transferred into new genera. The resulting monophyletic set of species would contain the type *A*. *alpina* together with ~20 additional species that are: (i) *A*. *alpina* and its closest relatives (~10–12 species), (ii) its sister clade including *A*. *nordmanniana* (*A*. *nordmanniana* clade; five species), and (iii) the *A*. *auriculata* clade (three species; Karl & Koch, 2013; Kiefer et al., 2017). Among those closest relatives of *A*. *alpina* are the red or pink flowering taxa *A*. *purpurea*, *A*. *cypria* and *A*. *aubrietoides*. Moreover, *A*. *deflexa*, *A. ionocalyx*, *A*. *caucasica* and *A*. *tianchanica* (Kyrgyzstan) are found within the same phylogenetically unresolved clade. If all of those were included within *A*. *alpina* to establish monophyletic entities, subspecies concepts would have to be applied (Karl et al., 2012).

*Arabis caucasica* has also been introduced in synonymy as *A*. *alpina* subsp. *caucasica* (additional synonyms are *A*. *albida*, *A*. *billardieri*; Koch et al., 2018). Although taxonomically accepted, there is no supporting genetic, geographical or morphometric information. The same applies to *A*. *alpina* subsp. *brevifolia*, which has been described from Eastern Mediterranean areas (Greuter & Raus, 1983), but also without any further complementary details. Consequently, there is no reliable and convincing infraspecific taxonomic system for *A*. *alpina* to date, and further taxonomic work is needed to correctly relate morphological variation with genetic make‐up and biogeographical patterns.

The entire species assemblage probably originated in the Pleistocene (Karl & Koch, 2013), and the three remaining species or taxa (*A*. *montbretiana*, *A*. *nova* subsp. *iberica*, *A*. *kennedyae*) comprise a well‐defined sister clade of exclusively annuals (Karl & Koch, 2013) that provide an excellent source for comparative evolutionary research (Kiefer et al., 2017, 2019). This sister clade split in the Late Pliocene or at the onset of the early Pleistocene glaciation/deglaciation cycles (Karl & Koch, 2013).

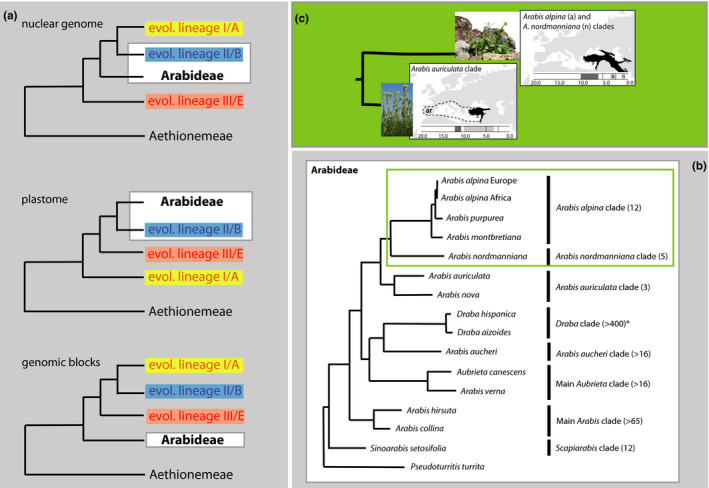

FIGURE B1 (a) Different phylogenetic positioning of tribe Arabideae within Brassicaceae core groups depending on the marker set used, indicating a biological phenomenon rather than an analytical artefact (following Walden, Nguyen, et al., 2020). (b) Summary of present‐day phylogenetic knowledge on clade relationships in Arabideae (species number given). Clade definition follows Karl and Koch (2013) and Kiefer et al. (2017). (c) Ancestral areas of the clade including *Arabis alpina* and its sister clades, highlighting the “Irano‐Turanian region” as a source of origin. The timeline indicates stem and crown group ages (including confidence intervals; Karl & Koch, 2013)

Phylogeographical studies on chloroplast and nuclear DNA indicate that *A*. *alpina* originated in Anatolia. The present‐day distribution was established with three ancestral lineages (Ansell et al., 2011; Koch et al., 2006) that diverged about 2–2.7 million years ago, at the Pliocene–Pleistocene transition. This period was marked by rapid cooling (Webb & Bartlein, 1992) and the expansion of habitats suitable for alpine plants. During the Pleistocene, a fragmented network of local survival centres persisted in Anatolia, possibly undergoing local elevational migrations during fluctuations between warmer interglacial and colder glacial periods (Ansell et al., 2011).

From Anatolia, a first lineage migrated to the Caucasus and the Iranian Plateau through the Anatolian diagonal. This high‐elevation mountain system probably provided stepping‐stone habitats for *A*. *alpina* to eventually reach the East African high mountains (Ansell et al., 2011; Koch et al., 2006). Within this lineage, the populations of the Anti‐Taurus and Mount Lebanon ranges form an independent clade (Ansell et al., 2011). A second, more southern lineage formed two phylogeographical groups in Ethiopia, which probably resulted from previously isolated populations that came into secondary contact with the East African lineage (Assefa et al., 2007; Koch et al., 2006). From Western Anatolia, a third lineage gave rise to all central and northern European populations through multiple immigration events, and also served as a source for the populations in Northwest Africa (Koch et al., 2006). Migration to Europe probably occurred through the region around the Sea of Marmara during colder glacial periods, when alpine habitats were located at lower elevations (Ansell et al., 2011). Within this third lineage, populations of *A*. *alpina* in the Alps and the Carpathians show high levels of overall genetic diversity and form a mosaic of differentiated groups with an east–west spatial structure (Alvarez et al., 2009; Ehrich et al., 2007). This pattern might result from multiple recolonizations from different refugia around and possibly within the Alps, the Carpathians and the Tatras (Ansell et al., 2008; Ehrich et al., 2007; Rogivue et al., 2018). Here, future phylogenomic approaches with sufficient numbers of genetic markers might provide conclusive evidence.

In more remote regions with milder climate, such as the Pyrenees and the Mediterranean, *A*. *alpina* might have persisted in situ during glacial periods (Ehrich et al., 2007). Northern European and North American populations, by contrast, show very low levels of genetic diversity (Ehrich et al., 2007). The authors propose colonization from a single refugium in Europe. However, this scenario appears unlikely given the vast periglacial area that expanded between the northern edge of the Alps and the northern European glaciers. Alternatively, multiple migrations with strong selection for colonization ability might have led to a selective sweep decreasing genomic diversity (Ehrich et al., 2007). Laenen et al. (2018) showed that the reduced genomic diversity of Scandinavian populations can be the consequence of a strong bottleneck associated with colonization from central Europe; however, their study could not resolve the question of single vs. multiple origin because it did not include enough samples from central Europe.

Detailed knowledge on the spatial genetic structure and the underlying demographic history is a valuable basis for investigating hypothesis‐driven questions in ecological genetics. Likewise, knowledge of neutral genetic structure is essential when inferring signatures of selection, because genomic imprints of past demographic processes at neutral loci, such as genetic drift, may mimic selective processes. Corresponding analyses require solid genomic resources, and a major step in this direction was the establishment of a high‐quality reference genome for *A*. *alpina*, as described below.

## 3 REFERENCE GENOME AND APPLICATIONS

With 475 Mbp, the genome of *Arabis alpina* is roughly 3.5 times the size of that of *Arabidopsis thaliana* (Willing et al., 2015). This difference in genome size largely relates to the accumulation of retrotransposons in both hetero‐ and euchromatic regions of the genome, exceeding that of other Brassicaceae species (Willing et al., 2015). Usually, transposable elements are not randomly distributed across a genome, but often accumulate within pericentromeric regions (e.g., The Arabidopsis Genome Initiative, 2000). In *A*. *alpina*, an evolutionarily recent transposition burst of *Gypsy* elements has led to the expansion of pericentromeric regions (Willing et al., 2015).

Pericentromeric regions can be characterized by low meiotic recombination rates and, hence, expansion of pericentromeric regions can alter the recombination landscape (Tanksley et al., 1992). In natural *A*. *alpina* populations within the Swiss Alps, transposon density was shown to be correlated with patterns of linkage disequilibrium along all chromosomes, and a large proportion of the linked blocks showed signatures of selective sweeps; those regions were enriched for genes that are underlying adaptive traits, which implies that transposon‐mediated genome dynamics play a key role in natural selection (Choudhury et al., 2019). Together with the fact that a large proportion of the gene space of *A*. *alpina* is contained in the heterochromatic compartment of the chromosome (Willing et al., 2015), those genomic features might complicate future genetic mapping experiments, which depend on segregation (i.e., breaking up of linkage groups), to identify causal polymorphisms that underlie signatures of selection or phenotype differentiation. Here, the obvious challenge with reduced recombination is that genes that are located within linked blocks cannot easily be isolated, so that a potential effect on the phenotype remains elusive. This can cause problems in resolving candidate loci that were identified by outlier scans (Buehler et al., 2014; Lobréaux & Miquel, 2020), but is equally problematic when segregating F_2_ populations are evaluated in common garden experiments (Toräng et al., 2017), where the number of plants that can be tested is often much lower than what would be needed according to recombination frequency. Finding solutions to these experimental issues will be important for a broad variety of species. Moreover, future research projects may be proposed that address the evolutionary history and functional implications of pericentromer expansion in *A*. *alpina* as a model system.

The most recent release of the reference genome and additional resources can be accessed at www.arabis‐alpina.org (Jiao et al., 2017). Resequencing of 35 individuals from across the species range (Laenen et al., 2018) and 304 from the western Swiss Alps (Rogivue et al., 2019) represent outstanding examples for sequencing of natural plant populations, and the publicly available data offer opportunities for future population genomic analyses. Additional genomic resources for *A*. *alpina* include the whole‐chloroplast genome sequence (Melodelima & Lobréaux, 2013) and a more fragmented reference genome assembly using individual and pooled‐sample sequencing of a Swiss population (Rellstab et al., 2020).

Within the Arabideae, morphological characters are frequently homoplastic (i.e., often reflect repeated gain and loss of character states), rendering the genus a fascinating system to investigate the molecular and genomic basis of, for example, parallel vs. convergent evolution. Consequently, future studies will benefit from additional sampling of selected species pairs along the phylogeny of the tribe. Moreover, phylogenomics approaches could be performed in the genus *Arabis* to decipher evolutionary trajectories in cytogenetic backgrounds complementary to the genus *Arabidopsis*, where such approaches have shed light on processes, such as ancient gene flow, that shaped the genomes of contemporary diploid species (Novikova et al., 2016). Here, one could, for example, include sister species of *A*. *alpina*, such as the annual *A*. *montbretiana* (Kiefer et al., 2017) with its much smaller genome, and the perennial, polyploid *A. nordmanniana*, allowing, for example, interspecies genome‐wide association studies (Kiefer et al., 2019). Moreover, only recently a study of the genomic properties of *A*. *sagittata* and *A*. *nemorensis* illustrated how genomic resources can be used for interspecific comparison and for tracing, for example, environmentally driven diversification and also extinction (Dittberner et al., 2019). Together, the series of insightful studies reviewed above illustrate applications of the *A*. *alpina* reference genome as a resource, and also provide directions for future research on the genome itself.

## 4 MATING SYSTEM EVOLUTION

Understanding the processes governing mating system evolution is pivotal, because the mating system by large influences the distribution of genetic diversity (Hamrick & Godt, 1996) and, thus, opportunities for adaptive evolution (Charlesworth, 2006; Hartfield et al., 2017). One ubiquitous pattern among angiosperm plants is the repeated transition to self‐compatibility and increased selfing (Barrett, 2002), which is commonly observed also in the Brassicaceae (Mable, 2008).

Earlier studies on the genetic structure of *A*. *alpina* found that the *F*_IS_ values of populations in the Alps were higher than in the Apennines, suggesting a difference in the mating system of alpine populations, specifically the regional absence of a functional self‐incompatibility system (Ansell et al., 2008). This hypothesis was tested through pollen tube visualization and sequencing of SRK genes, which confirmed a functional genetic barrier preventing individuals within the outcrossing populations in the Apennines from self‐fertilization through sporophytic self‐incompatibility, which has partly lost its function in the self‐compatible populations in the Alps (Tedder et al., 2011). As a consequence, populations in the Alps have lower outcrossing rates and lower proportions of self‐incompatible phenotypes than in the Apennines, as was shown by controlled pollination experiments (Tedder et al., 2011) and paternity analyses (Buehler et al., 2012).

Although these earlier studies characterized the genetic basis of the breeding system and confirmed its role in shaping patterns of genetic variation, they could not determine when self‐compatibility originated. Ansell et al. (2008) hypothesized that the self‐incompatibility system was lost when the species reached the Alps during postglacial recolonization from southern Italian refugia. Indirect evidence of the role of postglacial recolonization history in the evolution of the mating system might be the differences observed among self‐compatible populations: plants growing in the north (Scandinavia) show higher rates of self‐pollination, and consequently self‐fertilization, and *F*_IS_ values than plants from southern sites (France and Spain; Toräng et al., 2017). Using a demographic model fitted to single‐nucleotide polymorphism (SNP) data, Laenen et al. (2018) showed that the colonization of Scandinavia might have occurred between 20,000 and 24,000 years ago, but the timing of self‐incompatibility loss in Italy remains unknown, and it is still unclear whether self‐compatibility has also evolved in other places. The phylogeographical history of the species (see above) provides interesting opportunities to compare the selfing lineages of Europe, and potentially also of Africa, which are likely to represent independent breakdowns of self‐incompatibility.

Self‐compatible populations were used to study the evolution of morphological and physiological traits related to selfing (the selfing syndrome). Plants from self‐compatible populations have markedly smaller and less scented flowers, less herkogamy (anther–stigma distance) and lower pollen production than plants from outcrossing populations (Petrén et al., 2021; Tedder et al., 2015). In addition, anther orientation and herkogamy correlate with the efficiency of autonomous self‐pollination and pollen limitation, and hence they synergistically affect reproductive success. Therefore, these two traits can be crucial for the evolution of efficient self‐pollination and reproductive assurance in environments with low pollinator activity (Toräng et al., 2017). However, these studies could not assess the contribution of reproductive success to lifetime fitness, which also depends on germination, survival and growth. Comparisons of population growth rates (equivalent to mean fitness) between natural populations in the French Alps showed that differences in reproductive success and fecundity contributed less to differences in population growth rates than did differences in individual survival and growth rates (Andrello et al., 2020). While this is expected for long‐lived plants (Franco & Silvertown, 2004), these comparisons were all performed between self‐compatible populations and should be expanded to self‐incompatible populations in future studies.

In conclusion, studies of the reproductive system have highlighted *A*. *alpina* as a plant in which self‐pollination evolved during the postglacial colonization of its present range, and populations with varying degrees of selfing have allowed the study of the morphological and physiological traits related to self‐pollination. To understand the adaptive consequences of mating system variation, reciprocal transplant experiments could be done with self‐compatible and self‐incompatible plants, assessing their lifetime fitness in different abiotic and biotic environments. Future work could take advantage of the genomic resources of *A*. *alpina* to dissect the molecular mechanisms controlling the expression of herkogamy, anther orientation and other reproductive traits to further understand the genetic constraints on the evolution of selfing. In parallel, studies of the entire life cycle of plants growing in their natural environment are necessary to further characterize the selective pressures under which selfing is expected to evolve.

## 5 ADAPTATION TO ARCTIC AND ALPINE ENVIRONMENTS

Alpine environments are ideal settings for studies on local adaptation due to large habitat diversity over short distances (Scherrer & Körner, 2011), and patterns of differentiation were found in alpine plants along geographically narrow gradients (Byars et al., 2007; Kim & Donohue, 2013; Leempoel et al., 2018). The broad distribution of *Arabis alpina* offers many opportunities to investigate adaptive processes at different spatial scales. Habitats located on a latitudinal gradient from the East African mountains across the central European mountain chains to Scandinavia are expected to share typical features, such as a low annual mean temperature and a short growing season, while photoperiod length, but also light intensity, differ strongly. Habitats located in the Cantabrian mountains, the Pyrenees, the Central Alps and the Carpathian Mountains share aspects of alpine habitats, but differ in the degree of continentality. These clines can be exploited to test for broad‐scale natural variation in *A. alpina*, even though potential differences in mating systems may complicate disentangling the underlying causes. Moreover, local studies on adaptive responses to environmental contrasts can be replicated to test for convergent evolution or the contribution of biogeographical history to these selective processes. Consequently, *A*. *alpina* is an appropriate model system for studies on local adaptation and has become a showcase for landscape genomic analyses, though the full environmental range has yet to be exploited.

### 5.1 Experimental approaches

A classical long‐distance reciprocal transplantation experiment indicated local adaptation to lower winter minimum temperature in Scandinavia and more pronounced intensity of summer drought in Spain (Toräng et al., 2015). Significant signatures of local adaptation were also detected on a regional scale within the French Alps (de Villemereuil et al., 2018). Two common gardens hosting plants from six populations revealed phenotypic differentiation in traits related to both vegetative and reproductive performance: plants originating from low‐elevation sites grew more vigorously and had higher reproductive output than those from high‐elevation populations, which may relate to respective higher average temperatures and longer growing seasons. Such a trend in fitness across elevational gradients is commonly observed in experiments using plants (Halbritter et al., 2018), even though one should consider that other environmental factors besides temperature covary with elevation and may, thus, be among the ultimate drivers of such patterns. Yet larger experimental setups to involve numerous accessions of *A*. *alpina*, spanning a wide range of environmental conditions at source locations and replicated common gardens as in *Arabidopsis thaliana* (Fournier‐Level et al., 2011; see also https://grenenet.wordpress.com/), possibly including different treatments (Exposito‐Alonso et al., 2018), could help disentangle the various drivers of local adaptation patterns. With the genomic resources in *A*. *alpina* at hand, one could investigate the functional basis of the adaptive trait differences using large‐scale genome‐wide association analyses.

In the study of de Villemereuil et al. (2018), the degree of phenotypic plasticity depended on the population of origin, with plants from higher elevations showing less plasticity for the measured traits. Phenotypic plasticity was also detected in the comparison of Scandinavian and Spanish accessions (Toräng et al., 2015): the onset of flowering differed markedly between local and foreign populations at the Scandinavian site, but not so much at the Spanish site. Hence, it is likely that adaptation on both the large and regional scales includes specific cues that are only partially fulfilled in the foreign transplantation site. Given the latitudinal distance between Spain and Scandinavia, it might be expected that temperature interacts with photoperiod to synchronize flowering phenology with regional conditions (King & Heide, 2009). Testing for similar patterns by studying the effect of temperature differences at smaller spatial scales (low vs. high elevation), or along longitudinal clines (e.g., Pyrenees vs. Carpathian Mountains, Eastern vs. Western Alps) to hold photoperiod constant, might single out effects of gradients in climate and photoperiod and inform about their interactions.

The described studies on local adaptation in *A*. *alpina* focused on abiotic drivers and traits that show variation in relation to latitudinal and elevational contrasts. In the single study on adaptation to biotic drivers in *A. alpina*, Buckley et al. (2019) combined field observations and climate chamber experiments using populations from elevational clines to investigate variation in traits related to growth and herbivore defence. The authors found a link between herbivore pressure, leaf traits and defence compounds, which they associated with elevation‐driven phenotype combinations that are characterized by trade‐offs between growth and herbivore defence. Moreover, signals of adaptation were site‐specific even among populations located at the same elevation. These findings point towards complex interactions between abiotic and biotic factors that are largely undisclosed and pending further investigation. *Arabis alpina* as a common, widespread and easily cultivated plant species seems an attractive model for such field experiments that may teach us about trade‐offs between growth and herbivory.

### 5.2 Landscape genomics

*Arabis alpina* ranked among the first plant species used in landscape genomic studies involving outlier identification or environmental association analyses (also termed gene–environment analyses) for its wide‐ranging occurrence and broad ecological niche (see Box 1). Earlier studies attempting to identify genomic signals of local adaptation relied on genome scans using amplified fragment length polymorphisms (AFLPs). In *A. alpina*, outlier AFLP loci, considered to be adaptive due to significantly higher genetic differentiation than others, indicated temperature and precipitation as the main environmental drivers of local adaptation in several studies (Manel et al., 2010; Poncet et al., 2010; Zulliger et al., 2013). In two cases, such outlier loci were sequenced and found to be located within gene regions (Buehler et al., 2013; Zulliger et al., 2013), indicating potentially functional relevance. However, by genotyping an independent set of populations, Buehler et al. (2014) could not confirm respective alleles to associate with contrasting habitat types as in the original study (Buehler et al., 2013). This finding suggests that local adaptation to similar environmental cues may be compromised by extensive linkage disequilibrium, so that the causal genetic variation is not directly targeted, or involve different genes or gene networks in different sets of populations (Rellstab et al., 2017). Evidently, outlier analyses in anonymous markers as in these initial studies may be insufficient to resolve functionally relevant genes, while possibly pointing at candidate genomic regions for signatures of adaptation. Alternatively, local adaptation is indeed local, with independent genetic and functional responses to the same environmental cues even at small geographical scale.

More recent studies made use of whole‐genome sequencing data to find genomic imprints of selection. A genome scan based on reduced representation sequencing data identified a promising candidate gene which could explain differences in height and growth in *A*. *alpina* and is associated with growth performance in *Arabidopsis thaliana* (de Villemereuil et al., 2018). An additional 19 genomic regions were found to be associated with temperature, precipitation and snow cover (Lobréaux et al., 2014; Lobréaux & Miquel, 2020), and these included orthologues of genes involved in abiotic stress responses and the regulation of flowering in *Arabidopsis thaliana*. By increasing both genomic and spatial resolution, Rogivue et al. (2019) identified candidate SNPs for local adaptation related to stress and herbivore pressure. Interestingly, this study also identified transposable elements largely shared among populations and corresponding to temperature response terms. Finding gene–environment associations that also match the candidate gene annotation that has been inferred from the well‐studied *Arabidopsis thaliana* further strengthens such inference. Ultimately, it will be necessary to substantiate the functional relevance of gene variants on the basis of fitness measures under experimental conditions or by transgenic complementation approaches.

In an alpine environment, similar environmental factors are relevant for many plant species (Körner, 2003; Manel et al., 2012). It is hence possible that selection targets the same genes or pathways in phylogenetically distant species that occupy similar ecological niches (Stern, 2013), even though this general assumption may not hold true (see above). To date, two genome‐wide studies have compared the genes involved in adaptation to the alpine and arctic environment in different Brassicaceae species including *A*. *alpina* and found that adaptation comprised similar functional pathways, but most adaptive genes remained species‐specific (Birkeland et al., 2020; Rellstab et al., 2020). It will require many more such comparative studies before conclusive patterns of adaptive signatures across species and even genera may emerge.

In summary, it appears that common environmental drivers, such as temperature and precipitation, are associated with adaptive differentiation in *A*. *alpina*. While most of the discussed regional and local findings were uncovered within the French and Swiss parts of the European Alps, it remains to be tested if similar patterns exist elsewhere, taking full advantage of the broad distribution range of *A*. *alpina* and including populations that have evolved within tremendously different environments, such as in the East African mountains, or areas where outcrossing predominates.

## 6 POPULATION DYNAMICS AND DEMOGRAPHY

The demographic dynamics of natural populations contribute to shaping and partitioning natural variation within and among populations at the local and regional scale. Over 6 years, Andrello et al. (2020) studied demographic parameters (survival probability, growth variables, reproduction probability, fecundity) of individuals of *Arabis alpina* in six natural sites representing the species' elevational range in the Alps. The authors found mostly consistent values for mean annual survival probability (*S* = 0.5) and mean probability of reproduction (*F*_0_ = 0.5) across populations. Moreover, demographic rates were related to environmental conditions in the populations: with elevation, survival tended to increase, while growth and fecundity tended to decrease. These findings in *A. alpina* reflect common patterns in life histories of herbaceous plants: small, long‐lived species tend to inhabit high elevations, and vice versa (Laiolo & Obeso, 2017; Nobis & Schweingruber, 2013).

Despite marked clines in demographic rates along elevation, *A*. *alpina* showed surprisingly little variation in population growth rates across the large elevational range evaluated (Andrello et al., 2020). This lack of spatial variation could be partly ascribed to demographic compensation among different life‐cycle components (Andrello et al., 2020). In particular, increased survival probabilities at higher elevation compensated for lower fecundity. Moreover, interannual variation in survival probabilities, which reduces population growth rates, was smaller at high than at low elevation and further contributed to compensation. Such negative correlations between life‐cycle components can result from opposite responses to shared environmental drivers or from trade‐offs between different life‐cycle processes (Knops et al., 2007; Williams et al., 2015).

In addition to showing little spatial variation, population growth rates were negative in all sites, suggesting that populations are declining and might eventually go locally extinct (Andrello et al., 2020). Population growth rates were highly sensitive to survival probabilities and plant growth rates, whose relatively low values thus seem to be the proximate causes of such dynamics. However, the environmental factors leading to low values in these vital rates were far from understood, especially for survival probabilities (Andrello et al., 2020). There is thus ample space to further investigate the environmental drivers of population dynamics *in situ* (Ehrlén et al., 2016). In addition, the interdependency between flowering rates, shoot survival and whole‐plant senescence (see next section) makes *A*. *alpina* an excellent model system to study the effects of perenniality at the population level.

The high extinction probability of local populations agrees with *A*. *alpina* being a pioneer species with fast life histories that colonizes sites after disturbance and when conditions are favourable for germination (Lopez del Egido et al., 2018). Hence, the fast turnover of local populations might be the outcome of extinction–colonization processes at the metapopulation level, but the observed strong spatial genetic differentiation (de Villemereuil et al., 2018) may alternatively imply low seed dispersal rates. Colonization of empty sites can occur via germination from a persistent seed bank, since viable seeds of *A*. *alpina* were observed in several arctic–alpine soils (Diemer & Prock, 1993; Philipp et al., 2018). Metapopulation theory has received comparatively little attention in plant studies, partly because the long lifespan of many plant species and apparent stability of their populations make it hard to document true extinction and recolonization events (Ouborg & Eriksson, 2004). Conversely, due to its relatively short lifespan, pioneer strategy and observed local population declines, we consider *A*. *alpina* an attractive model species to study plant population dynamics under the lens of metapopulation theory.

## 7 PERENNIAL GROWTH HABIT AND TRAIT EVOLUTION

Polycarpic plants such as *Arabis alpina* can flower and reproduce several times during their lifetime, whereas monocarpic plants are commonly annual and reproduce only once before they die (for details on modes of parity see Hughes, 2017). Within the Brassicaceae, the evolutionary transition from polycarpic to monocarpic life history has occurred many times (Kiefer et al., 2017), providing multiple opportunities for comparative studies. For example, the molecular mechanisms that contribute to life‐history evolution can be dissected between *A*. *alpina* and its annual relatives *A*. *montbretiana* and *Arabidopsis thaliana*. Below, we showcase some important traits that contribute to the polycarpic life history of *A*. *alpina*, describe their molecular underpinnings, and put them into an ecological context.

### 7.1 Juvenile phase

Polycarpic plants often undergo a juvenile phase, during which they cannot flower even upon receiving the appropriate environmental cues that would induce flowering of adult individuals (Hyun et al., 2017). Seedlings of the Spanish *A*. *alpina* reference accession Pajares require a minimum of 5 weeks of growth in controlled long‐day conditions before they are competent to respond to flower‐inductive stimuli (Wang et al., 2011). The molecular mechanisms regulating juvenile–adult phase change in *A*. *alpina* are similar to those described in *Arabidopsis thaliana*, where this trait is regulated by the sequential action of two microRNAs, miR156 and miR172 (Wu et al., 2009). Typically, miR156 accumulates in the apices of young seedlings and decreases as plants become older and reach reproductive maturity, while at the same time levels of miR172 increase (Wu et al., 2009). miR156 targets a family of SQUAMOSA PROMOTER‐BINDING PROTEIN‐LIKE (SPL) transcription factors, whereas miR172 regulates a subclade of AP2 transcription factors which include APETALA2 (AP2) and the AP2‐like SCHLAFMÜTZE (SMZ), SCHNARCHZAPFEN (SNZ) and TARGET OF EAT1‐3 (TOE1‐3; Aukerman & Sakai, 2003; Wang et al., 2009; Wu et al., 2009). Orthologues of some of these genes have been functionally characterized in *A*. *alpina* and it has been demonstrated that they equally contribute to the age‐dependent control of flowering. These factors include the *A*. *alpina* orthologues of *SQUAMOSA PROMOTER*‐*BINDING PROTEIN*‐*LIKE15* (*AaSPL15*), *PERPETUAL FLOWERING2* (*PEP2*, the *A*. *alpina* orthologue of *AP2*) and *TARGET OF EAT2* (*AaTOE2*; Bergonzi et al., 2013; Hyun et al., 2019; Lazaro et al., 2019; Zhou et al., 2021). While the role of these genes in the age pathway is conserved between *Arabidopsis thaliana* and *A*. *alpina*, they additionally contribute to polycarpy of *A*. *alpina* (Table 1). For example, the maintenance of vegetative growth after flowering is compromised in *pep2* and *toe2* mutants, and in transgenic lines that express microRNA‐resistant forms of AaSPL15, in which all axillary branches become reproductive (Table 1; Hyun et al., 2019; Lazaro et al., 2019; Zhou et al., 2021). Compared to monocarpic *Arabidopsis thaliana*, these additional roles of age‐related factors in polycarpic *A*. *alpina* may reflect a more prominent role of the age pathway that cannot be bypassed by other flowering‐time pathways (Hyun et al., 2017, 2019). This would help prevent plants from flowering precociously before they have crossed a potential physiological threshold that permits vegetative perseverance after reproduction.

**TABLE 1:** Conserved and diverse roles of genes functionally characterized in *Arabis alpina* (Aa) compared to *Arabidopsis thaliana* (At)

Gene (At)	Gene (Aa)	Trait (At)	Trait (Aa)	Reference
FLC	PEP1	Flowering, seed dormancy	Flowering, polycarpy, duration of flowering episode, seed dormancy and longevity	Hughes et al. (2019), Lazaro et al. (2018), Wang, Farrona, et al. (2009)
TFL1	AaTFL1	Flowering	Age‐dependent response to vernalization, polycarpy	Wang et al. (2011)
SPL15	AaSPL15	Flowering	Age‐dependent response to vernalization, polycarpy	Hyun et al. (2019)
AP2	PEP2	Flowering	Age‐dependent response to vernalization, polycarpy	Bergonzi et al. (2013), Zhou et al. (2021)
TOE2	AaTOE2	Flowering	Age‐dependent response to vernalization, polycarpy	Zhou et al. (2021)
BRC1	AaBRC1	Bud dormancy	Bud dormancy	Vayssières et al. (2020)
TTG1	AaTTG1	Trichome and root hair formation	Trichome and root hair formation	Chopra et al. (2014)
GL2	AaGL2	Trichome patterning	Trichome patterning	Chopra et al. (2019)

### 7.2 Flowering behaviour

Cold is a major environmental factor that plants have to cope with, but at the same time it provides a distinct cue for synchronizing flowering with favourable environmental conditions. The *A*. *alpina* reference accession Pajares from Spain requires exposure to prolonged cold to flower, a process commonly known as vernalization. Arctic–alpine species experience additional constraints from their environment, which is characterized by long winters and unpredictably short growing seasons. Hence, many plants from such habitats initiate floral organs several months or even years before anthesis, so that they can flower rapidly once environmental conditions are benign (Billings & Mooney, 1968; Diggle, 1997; Körner, 2003). In controlled experimental conditions, *A*. *alpina* flower buds are formed during cultivation at 4°C (Lazaro et al., 2018; Wang, Farrona, et al., 2009) and in natural populations, flower buds are already present in autumn (S. Wötzel, personal observation), suggesting that *A*. *alpina* plants can be vernalized and initiate flowering before winter. Moreover, experiments characterizing the flowering response of the reference accession Pajares to different durations of cold treatment indicated that exposure to less than 12 weeks of cold leads to asynchronous flowering among the assayed individuals and compromised floral organ identity, thereby reducing reproductive output (Lazaro et al., 2018; Wang, Farrona, et al., 2009). These results suggest that natural populations may require locale‐specific cold periods for optimal reproduction, a hypothesis that could be tested by evaluating the response of several natural accessions to different cold treatments.

### 7.3 Polycarpy

Flowering in response to vernalization in *A*. *alpina* is mediated by the MADS box transcription factor PERPETUAL FLOWERING 1 (PEP1; Wang, Farrona, et al., 2009), which is the orthologue of *Arabidopsis thaliana FLOWERING LOCUS C* (*FLC*; Michaels & Amasino, 1999; Sheldon et al., 2000). Similar to *FLC*, *PEP1* acts as a floral repressor and is transcriptionally regulated by vernalization. In *A*. *alpina* plants of the reference accession (wild‐type), *PEP1* transcript abundance decreases during cold exposure and plants form flower buds (Lazaro et al., 2018; Wang, Farrona, et al., 2009). In addition, *pep1* mutants and natural accessions with nonfunctional alleles of *PEP1* flower early and without cold exposure (Albani et al., 2012; Wang, Farrona, et al., 2009), resembling the early flowering phenotype of *Arabidopsis thaliana flc* mutants (Michaels & Amasino, 1999).

These findings confirm functional orthology, but vernalization stably silences *PEP1* only in mature meristems, which initiate reproductive development during cold exposure, but not in juvenile meristems, which remain vegetative (Lazaro et al., 2018). *PEP1* mRNA levels are elevated in meristems that did not commit to flowering during vernalization, which ensures the maintenance of vegetative growth in the following year (Lazaro et al., 2018; Wang, Farrona, et al., 2009). This suggests that besides the age pathway, differences in vernalization‐mediated *PEP1* stable silencing also contribute to the polycarpic growth habit of *A*. *alpina* (Table 1; reviewed in Soppe et al., 2021). In addition, PEP1 may pleiotropically contribute to the maintenance of basal dormant buds by ensuring the continuous supply of auxin from vegetative axillary branches and, hence, structuring the primary shoot even after reproduction (Ponraj & Theres, 2020; Soppe et al., 2021; Vayssières et al., 2020). Interestingly, accessions with nonfunctional *PEP1* alleles showed increased mortality in common gardens, suggesting that PEP1 function is relevant for plant survival (Albani et al., 2012; Hughes et al., 2019). This is probably related to the reduced vegetative perseverance observed in *pep1* mutants that readily flower on many branches, which in turn are no longer available for vegetative growth. In addition, seed longevity and dormancy were reduced in *pep1* mutants (Table 1; Hughes et al., 2019), suggesting some accessions may undergo a shift towards a rapid‐cycling life strategy that closely resembles the monocarpic growth habit.

In summary, the traits contributing to the perennial growth habit are increasingly well understood at the molecular level. However, future research might have to emancipate even further from monocarpic models to uncover how flowering of individual branches is orchestrated on an adult plant after first flowering, and how flowering and vegetative perseverance are balanced throughout. Considering the described relationship between axillary bud formation, juvenile phase length and the developmental fate of lateral branches, integrative approaches including phenologically linked traits will be well suited to investigate the principles of life history divergence.

### 7.4 Additional phenotypic traits

**FIGURE 2:**
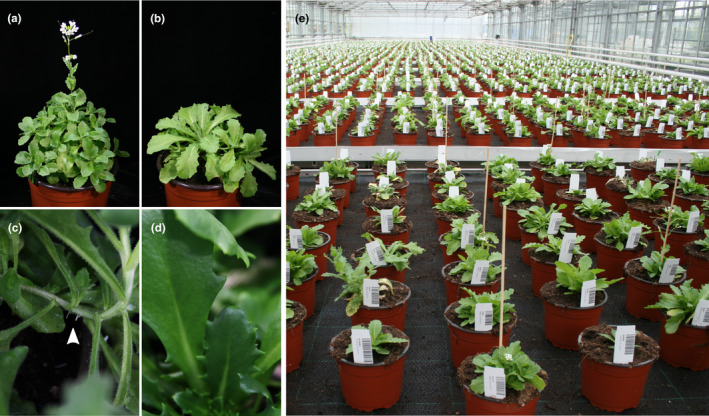
Examples of developmental traits studied using *Arabis alpina* (a–d): flowering time in the glasshouse varies between plants from the same population (a, b), adventitious rooting (arrow) at lateral branches (c), and a naturally occurring variant with low trichome density (d). Illustration of large‐scale glasshouse survey testing for naturally occurring variation in developmental traits (e) Photos: S. Wötzel

*Arabis alpina* has also been used to study further developmental traits (exemplified in Figure 2), which include leaf trichome formation (Chopra et al., 2014, 2019), leaf senescence (Wingler et al., 2012, 2015), secondary growth (Sergeeva, Liu, et al., 2021; Sergeeva, Mettler‐Altmann, et al., 2021) and adventitious rooting (Mishra et al., 2020). For the last of these, natural variation has been detected under controlled environmental conditions (Mishra et al., 2020). This finding might indicate a potential adaptive significance for this trait, with different phenotypic optima for individual populations. Observations in natural alpine environments suggest that adventitious roots in *A*. *alpina* may primarily serve for anchoring the growing plant body to unstable substrate (Figure 1h), an adaptation similar to those found in other scree‐inhabiting plant species (Körner, 2003).

In summary, comparative physiological studies between *A*. *alpina* and *Arabidopsis thaliana*, chemically induced mutants and transgenic lines, and the evaluation of natural accessions of *A*. *alpina* have resulted in a detailed understanding of polycarpic life‐history traits at the molecular level. Many, if not all, of these traits may be of adaptive significance, and the example of flowering time has highlighted the crucial role of naturally occurring variants for characterizing gene functions. Future molecular studies could target candidate loci that were identified by landscape genomics methods (see above) and provide the proof‐of‐concept data that are often the step remaining to be completed in contemporary studies of adaptation. Moreover, interspecific comparison of trait expression and gene function between mono‐ and polycarpic species within the Arabideae will provide insight into the mechanistic basis of life‐history evolution. One major difference between the two life‐history strategies that awaits further research is that senescence after flowering is restricted to reproductive branches in polycarpic plants, whereas it is global in monocarpic plants. To this end, introgression lines of *A*. *alpina* that contain genomic regions of the annual close relative *A*. *montbretiana* in a common genetic background have already been developed as a genetic tool (Hyun et al., 2019; Kiefer et al., 2017). It will be interesting to see to what extent the molecular pathways underlying relevant traits diverged and how these can be related back to the environments in which the contrasting life histories have evolved.

## 8 PERSPECTIVES

In this overview, we demonstrate that *Arabis alpina* is well suited to answer questions related to the adaptation of plants to harsh arctic–alpine environments and the evolution of a perennial life history. However, despite the progress of the past decade, there are still many open questions that await to be addressed. Below, we formulate some avenues that may guide future research in the fields of ecological, evolutionary and molecular genetics in *A*. *alpina* and other taxa. Advancement will greatly profit from interweaving the complementary approaches reviewed above, which benefit from the ample resources already available in this model species.

### 8.1 Adaptive physiology of stress resistance

While for some ecologically relevant and naturally variable traits, such as the timing of flowering, the molecular genetic basis is already well understood, other traits still await detailed molecular characterization, for example those regulating frost tolerance through the accumulation of metabolites in leaves (Kolaksazov et al., 2017). Storage compounds are deposited exclusively in the perennating parts of the plant that also exhibit secondary growth (Sergeeva, Liu, et al., 2021; Sergeeva, Mettler‐Altmann, et al., 2021). Some storage compounds, such as sucrose, confer higher tolerance to cold stress in plants from high elevations, while leading to accelerated leaf senescence in plants from low elevation (Wingler et al., 2012, 2015). Identifying the genes controlling sucrose accumulation will not only shed light on the molecular basis of frost resistance, but might also uncover genes with pleiotropic effects on senescence‐related traits. Similarly, traits conferring resistance to pathogen infections and herbivory were experimentally characterized (Buckley et al., 2019), but still await investigation at the genomic and molecular level to utilize our understanding beyond single‐factorial effects of certain genes and gene networks.

### 8.2 Mutualistic interactions

Another key to the adaptive success of plants is their ability to associate with mutualistic organisms. In many taxa, fungi or other microbiota grow on plant roots to form close symbioses. While mycorrhizal associations typically do not occur in Brassicaceae, *A*. *alpina* is an exception to this rule, but only limited information on how *A*. *alpina* may benefit from such root associates is available to date. For example, Almario et al. (2017) characterized fungal microbiota on *A*. *alpina* roots and found certain taxa to be dominant on soils with low phosphorus content. Their results indicate microbial support for phosphorus uptake under limiting conditions and suggest that taxa growing on alpine, often nutrient‐poor soils can evolve alternative solutions to the otherwise common mycorrhizal symbiosis. Nevertheless, it remains unclear how common these associations are, whether they lead to co‐adaptations in the involved physiological pathways, and to what degree they influence the evolutionary trajectory of the host plant and contribute to the ability of *A*. *alpina* to grow on poor soils as a pioneer species. Here, hypotheses could be developed in relation to the extended phenotype concept (Gugerli et al., 2013; Whitham et al., 2006) and tested using *A*. *alpina* as a species growing across a broad ecological niche, including gradients of elevation or nutrient availability (Buehler et al., 2013). Along this line, we advocate a shift from research targeting largely abiotic cues towards studies on biotic interactions and how these shape evolutionary processes, as exemplified in the next section.

### 8.3 Mating system and beyond

The mating system of plants largely evolves in relation to plant–pollinator interactions. Addressing the genetic basis of floral scent variation (Petrén et al., 2021), but also other traits related to mating system divergence (Toräng et al., 2017), will shed light on the genomics of mating system evolution in a phylogeographical context that relates to the species' demographic history. Disentangling the genetic architecture of floral scent production may show which of the genetic modules that encode different steps in the underlying biochemical pathways differentiate self‐compatible from self‐incompatible accessions. Did those modules evolve independently in different self‐compatible populations? Is there genetic linkage with genomic regions responsible for self‐compatibility? Does the extent of pericentromer expansion differ between self‐compatible and self‐incompatible accessions? How do chemical components of scent relate to the different pollinator guilds across the continents? Finding answers to such questions relies on connecting physiological, molecular, genomic and ecological approaches that integrate demographic inference, for all of which *A*. *alpina* already provides a solid foundation to build on.

### 8.4 Broadening the (phylo)geographical landscape

While most of the reviewed research on *A*. *alpina* has so far centred on populations from the European Alps, Scandinavia and the Spanish reference accession Pajares, future studies should expand along the species' phylogeography. Accessions from the Anatolian mountains and African sky islands, which represent isolated high‐elevation habitats for *A*. *alpina* in today's tropical latitudes (Chala et al., 2017), might contain unexpected genetic variants underlying traits that provide adaptation to a strongly divergent environment. In particular, the equatorial daytime climate, to which the African populations are exposed, radically differs from the extreme seasonal habitats of northern latitudes. Moreover, analyses of such distinct lineages might change the understanding of the species' evolutionary history, similarly to what has been shown for the example of *Arabidopsis thaliana* after including previously neglected accessions from Africa (Durvasula et al., 2017). It is also possible that accessions from more southerly climates contain genetic variants that may provide adaptive advantages for cold‐adapted species that are threatened by global warming. We thus propose a more global perspective for future sampling and experimental work involving *A*. *alpina* to broaden the environmental perspective provided by such a wide‐ranging taxon.

### 8.5 Comparative genomics

Biological discoveries can be expanded from *A*. *alpina* to closely related sister species within the Arabideae, and to those in other well‐studied genera of the Brassicaceae such as *Arabidopsis*, *Capsella*, *Boechera*, *Cardamine* and *Brassica* (Krämer, 2015; Rushworth et al., 2011). With ever more plant genomes available, comparative genomics are becoming increasingly feasible and could, for example, specifically address adaptation to extreme environmental factors such as high irradiance, low nutrient content, low temperatures, persistent snow cover and short growing season that characterize arctic and/or alpine habitats. How similar is the genomic basis of evolutionary solutions to these ecological challenges in different species? Do different strategies exist, are differences found within the same genes or gene networks, or were complementary pathways involved in these adaptive processes? Do we find common gene clusters involved in adaptive responses to arctic–alpine habitats? Studies including different Brassicaceae species have just begun to address principles of adaptation to the environment (Birkeland et al., 2020; Nowak et al., 2021; Rellstab et al., 2020), or to whole‐genome duplication (Bohutínská et al., 2021).

Lessons learnt from an arctic–alpine model system may help pinpoint the genomic particularities that also govern physiological processes in other taxa under stressful conditions (Park et al., 2017). In return, it might be asked to what extent interspecific differences in life‐history and functional traits (Adler et al., 2014; Nobis & Schweingruber, 2013; Read et al., 2014) can be observed at the intraspecific level to broaden our knowledge on microevolutionary processes. One line of such research is directed towards the effects of variation in transposable elements (TEs) and epigenetics. Environmental stress has been shown, for example in *Arabidopsis thaliana*, to induce enhanced activity of TEs, which in turn affects gene integrity or the regulation of gene expression. Epigenetic transmission can then lead to an acquired adaptive response in the next generation (Thieme & Bucher, 2018; Thieme et al., 2017). While epigenetic studies are becoming increasingly prominent (Richards et al., 2017), we are unaware of any investigation that evaluates how epigenetic changes contribute to the ability of *A*. *alpina* to cope with environmental stressors. Here, we see great potential in establishing links between genome‐wide methylation patterns and the pertinent information on the types, activity and distribution of TEs (Choudhury et al., 2017, 2019).

## 9 CONCLUSION

Taken together, the marked variation in life‐history traits encountered across the natural range of *A*. *alpina*, together with the publicly available genomic resources established in recent years, will serve as a comprehensive basis for future studies in this model for ecological genomics and beyond. With our overview, we demonstrate that *A*. *alpina* is a valuable and integrative model system that can join different research fields in plant biology. Together with a suite of other taxa, *A*. *alpina* complements research in the genus *Arabidopsis*, on which a huge array of research still relies, to address alternative evolutionary routes to environmental cues. Additional, intensively studied taxa will enrich the field of evolutionary plant biology and contribute to knowledge about the manifold ways plants cope with their abiotic and biotic environment.

## AUTHOR CONTRIBUTIONS

S.W., M.A., M.C.A., G.C. and F.G. conceived the outline, S.W., M.A., M.C.A., M.A.K. and F.G. wrote the article with contributions from G.C., and all authors read and approved the final version.

## CONFLICTS OF INTEREST

The authors declare no conflicts of interest.

## Supporting information

Appendix S1

## Data Availability

Data are provided in Supporting Information.
